# Limitations of mouse models for sickle cell disease conferred by their human globin transgene configurations

**DOI:** 10.1242/dmm.049463

**Published:** 2022-07-07

**Authors:** Kaitly J. Woodard, Phillip A. Doerfler, Kalin D. Mayberry, Akshay Sharma, Rachel Levine, Jonathan Yen, Virginia Valentine, Lance E. Palmer, Marc Valentine, Mitchell J. Weiss

**Affiliations:** 1 Department of Hematology, St. Jude Children's Research Hospital, Memphis, TN 38105, USA; 2 Integrated Biomedical Sciences Program, College of Graduate Health Sciences, University of Tennessee Health Science Center, Memphis, TN 38163, USA; 3 Department of Bone Marrow Transplantation and Cellular Therapy, St. Jude Children's Research Hospital, Memphis, TN 38105, USA; 4 Cytogenetics, St. Jude Children's Research Hospital, Memphis, TN 38105, USA

**Keywords:** Fetal hemoglobin, Genome editing, Sickle cell disease

## Abstract

We characterized the human β-like globin transgenes in two mouse models of sickle cell disease (SCD) and tested a genome-editing strategy to induce red blood cell fetal hemoglobin (HbF; α_2_γ_2_). Berkeley SCD mice contain four to 22 randomly arranged, fragmented copies of three human transgenes (*HBA1*, *HBG2-HBG1-HBD-HBB^S^* and a mini-locus control region) integrated into a single site of mouse chromosome 1. Cas9 disruption of the BCL11A repressor binding motif in the γ-globin gene (*HBG1* and *HBG2*; HBG) promoters of Berkeley mouse hematopoietic stem cells (HSCs) caused extensive death from multiple double-strand DNA breaks. Long-range sequencing of Townes SCD mice verified that the endogenous *Hbb* genes were replaced by single-copy segments of human *HBG1* and *HBB^S^* including proximal but not some distal gene-regulatory elements. Townes mouse HSCs were viable after Cas9 disruption of the *HBG1* BCL11A binding motif but failed to induce HbF to therapeutic levels, contrasting with human HSCs. Our findings provide practical information on the genomic structures of two common mouse SCD models, illustrate their limitations for analyzing therapies to induce HbF and confirm the importance of distal DNA elements in human globin regulation.

This article has an associated First Person interview with the first author of the paper.

## Abstract (precis)

**Editor's choice:** This study describes the genomic structures of two common sickle cell disease mouse models, illustrates their limitations for analyzing some genetic therapies and confirms the importance of distal DNA elements in human globin gene regulation.

## INTRODUCTION

Sickle cell disease (SCD) is among the most common human monogenic disorders and the first one to be understood at the molecular level (Ingram, 1956; Kato et al., 2018; Pauling et al., 1949; Piel et al., 2017). Most affected individuals are homozygous for a β-globin gene (*HBB*) missense mutation (Glu6Val), resulting in a structurally abnormal hemoglobin tetramer (HbS; α_2_β^S^_2_). At low O_2_ tension, HbS forms stiff polymers that distort red blood cells (RBCs) into a sickle shape and trigger a pathophysiology of hemolysis, inflammation and vascular occlusion. Clinical consequences include severe acute and chronic pain, impaired immunity, progressive multi-organ damage and early mortality. Current medical therapies including antibiotic prophylaxis, immunizations, RBC transfusions and hydroxyurea, have reduced complications and extended the life expectancy of affected children, although most patients still experience severe morbidities and die prematurely. Allogeneic hematopoietic stem cell (HSC) transplantation from an HLA-matched sibling can cure SCD, with long-term disease-free survival rates of >95% (de la Fuente et al., 2020; Eapen et al., 2019; Gluckman et al., 2017; Hulbert and Shenoy, 2018). However, the procedure remains high risk and most patients lack optimal donors.

Recent insights into RBC biology combined with technological advances in manipulating the genome are now fueling the development of potentially curative SCD therapies based on the genetic modification of autologous HSCs (Naldini, 2019; Orkin and Bauer, 2019; Wienert et al., 2018). These approaches include lentiviral vector expression of an anti-sickling β-like globin (Kanter et al., 2021), genome editing or base editing to eliminate the SCD mutation (Dever et al., 2016; Hoban et al., 2016; Newby et al., 2021), and genetic manipulations to induce the expression of RBC fetal hemoglobin (HbF; α_2_γ_2_), which alleviates SCD by inhibiting HbS polymerization (Eaton and Bunn, 2017). Early clinical trial results demonstrate therapeutic induction of HbF by using Cas9-mediated non-homologous end joining (NHEJ) to disrupt an erythroid enhancer of the *BCL11A* gene, which encodes a transcriptional repressor that binds γ-globin gene promoters to inhibit their expression (Frangoul et al., 2021), or by using a lentiviral vector to express anti-*BCL11A* short hairpin RNA in erythroid precursors (Esrick et al., 2021). It is also possible to induce HbF by disrupting a key BCL11A binding motif in the γ-globin promoters, thereby recapitulating naturally occurring human hereditary persistence of fetal hemoglobin (HPFH) variants that are associated with elevated levels of RBC HbF and reduced or absent SCD manifestations (Liu et al., 2018; Martyn et al., 2018; Métais et al., 2019; Traxler et al., 2016).

Studies in mice have been instrumental for preclinical testing of new SCD therapies. For example, genetic modification of human HSCs to induce HbF in erythroid progeny can be evaluated by xenotransplantation into NBSGW immunodeficient mice (Brendel et al., 2016; Métais et al., 2019). However, this approach cannot be used to examine the end organ effects of SCD because both normal and SCD human RBCs are rapidly cleared from the mouse circulation (Hu et al., 2011). Many studies evaluating SCD pathophysiology and/or new therapies are performed using two strains of mice (Berkeley and Townes), which express human globin genes instead of the mouse paralogs (Pászty et al., 1997; Wu et al., 2006). Of note, the developmental regulation of endogenous β-like globin genes differs between species (Hardison, 2012). In mice, two major embryonic β-like globin genes, *Hbb-y* and *Hbb-bh1*, are expressed during early gestation (Hill et al., 1984; Kingsley et al., 2006). Expression of the adult genes *Hbb-b^maj^* and *Hbb-b^min^* begins during mid-gestation and continues throughout life. In humans, ε-globin (*HBE*; also known as *HBE1*) is expressed during early embryogenesis. Expression of the fetal γ-globin genes, *HBG1* and *HBG2*, begins in mid-gestation and declines around birth, as adult β-globin (*HBB*) becomes activated reciprocally (Sankaran and Orkin, 2013). Thus, mice lack a ‘fetal’ β-like globin gene that is expressed analogously to that of the human γ-globin genes.

The Berkeley SCD mouse harbors genetic disruptions of endogenous adult-type α- and β-globin genes (*Hbb-a1*, *Hbb-a2*, *Hbb-b^maj^* and *Hbb-b^min^*) and expresses human globins via three DNA transgenes: 1.5 kb spanning the α-globin gene *HBA1*; a contiguous 39 kb genomic fragment including genes for γ-globin (*HBG2*, *HBG1*), δ-globin (*HBD*) and sickle β-globin (*HBB^S^*); and a 6.5 kb *‘*mini-locus control region (LCR)’ derived from an endogenous enhancer in the human β-like globin cluster that confers high-level erythroid expression (Pászty et al., 1997). A different SCD model, commonly referred to as the Townes mouse, was generated by using homologous recombination to replace adult-expressed mouse α-globin genes with human *HBA1*, and adult-expressed mouse β-like globin genes with tandemly linked genomic segments of human *HBG1* and *HBB^S^* (Wu et al., 2006). The Townes and Berkeley mice have been used extensively for testing HSC genetic modifications to treat SCD, including lentiviral vector-mediated β-globin gene replacement and altering the mutant SCD codon via Cas9-induced homology-directed DNA repair or base editing (Newby et al., 2021; Pawliuk et al., 2001; Perumbeti et al., 2009; Pestina et al., 2009; Urbinati et al., 2018; Wilkinson et al., 2021). However, the capacity of these strains to assess therapeutic induction of RBC HbF may be more limited by the human transgene configurations and/or interspecies differences in gene regulation.

In this study, we characterized the human globin genes in Townes and Berkeley mice and examined the effects of CRISPR/Cas9-mediated disruption of the γ-globin BCL11A binding motif in hematopoietic stem and progenitor cells (HSPCs). Our findings provide new information on the transgene structures and identify distinct experimental limitations for each strain, including lethal genotoxicity associated with Cas9-induced double-strand DNA breaks in the multi-copy γ-globin transgenes of Berkeley mice and sub-physiological induction of HbF after disrupting the BCL11A binding motif in the *HBG1* gene in Townes mice.

## RESULTS

### Genomic characterization of the Berkeley SCD mouse

**Fig. 1.**
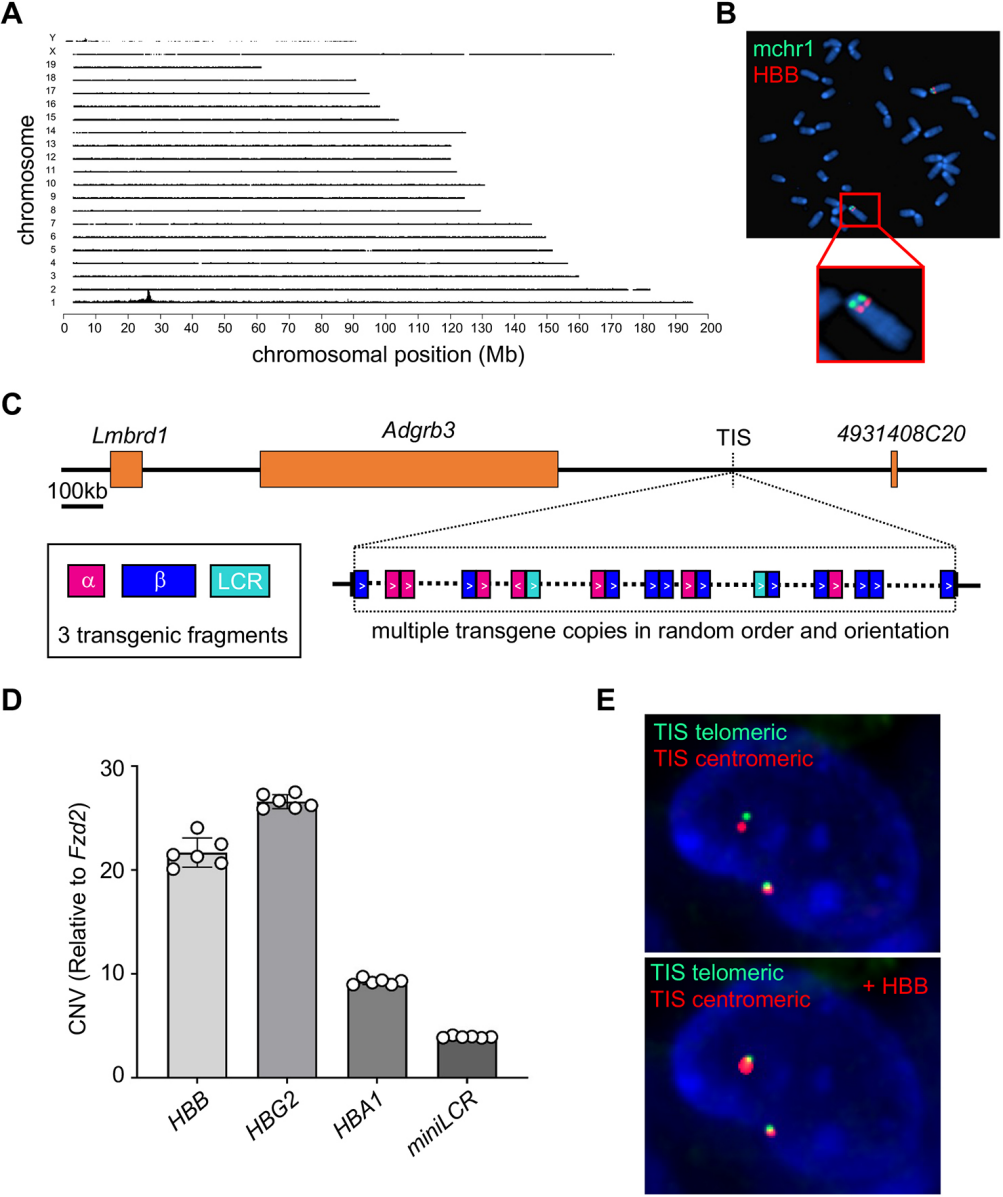
**Human globin transgene structure in the Berkeley mouse model for sickle cell disease (SCD).** Berkeley mice harbor three separate human transgenes encoding α-globin (*HBA1*; 1.5 kb), the extended β-like globin locus (*HBB^S^-HBD-HBG1-HBG2*; 39 kb) and a mini-locus control region (LCR; 6.5 kb), and lack endogenous mouse globin genes. (A) Target locus amplification (TLA) sequence analysis of each human transgene and flanking mouse genomic DNA was performed in a homozygous Berkeley mouse. Graph shows sequence coverage across the mouse genome. The peak signal on mouse chromosome 1 (mchr1) indicates a single integration site for all three human transgenes. (B) Metaphase fluorescence *in situ* hybridization (FISH) analysis of Berkeley mouse fibroblasts using probes for human *HBB* (red) and a control region of mchr1 (green). (C) Map of mchr1 near the transgene integration site (TIS). Human transgene configurations derived from a subset of TLA fusion reads is shown for *HBA1* (pink), *HBB* (blue) and mini-LCR (teal) fragments with directionality indicated by arrowheads. The diagram does not represent a precise order of integrated transgenes or include all copies. (D) Copy number estimation of *HBB*, *HBA1*, *HBG2* and mini-LCR by droplet digital PCR (ddPCR) of genomic DNA from Berkeley mice (*n*=6) heterozygous for the human transgenes, relative to the single-copy mouse gene *Fzd2*. (E) The top panel shows FISH images of heterozygous Berkeley mouse lung fibroblasts labeled with BAC clones flanking the transgene TIS (centromeric: RP23-308J23, red; telomeric: RP24-347O20, green). The bottom panel shows the same cell with the human transgene-containing chromosome labeled with an HBB probe (RP11-622D14, red).

We performed target locus amplification (TLA) sequencing on Berkeley mouse genomic DNA to characterize the human globin transgenes. This method employs crosslinking of physically proximal DNA sequences to selectively amplify and sequence genomic regions of interest (Fig. S1A) (De Vree et al., 2014; Hottentot et al., 2017). Most of the mouse-specific TLA sequences that were fused to human globin genes mapped to a single transgene integration site (TIS) in mouse chromosome 1 (Fig. 1A). A 663 bp segment of mouse chromosomal DNA (mchr1:26,274,199-26,274,861, mm10) was deleted at the TIS, presumably during transgene integration. The chromosome 1 TIS was confirmed by fluorescence *in situ* hybridization (FISH) analysis of Berkeley mouse lung fibroblasts using an *HBB* probe (Fig. 1B). The TIS is situated in a region of chromatin that is normally closed in hematopoietic cells, ∼400 kb from the nearest annotated genes (Fig. 1C; Fig. S1B). TLA sequencing coverage of the region in Berkeley mice identified multiple fusions of the three transgenic fragments in different orders, orientations and sizes (Fig. 1C). The ratio of sequencing coverage between the transgenic fragments and the mouse chromosome integration site on chromosome 1 was >100× for each of six primer sets used for TLA sequencing (Fig. S2), indicating that multiple copies of each transgene are present. However, it is not possible to estimate transgene copy number accurately with TLA analysis because the PCR primers preferentially amplify transgene sequences over mouse genomic DNA.

We estimated the copy number of each transgenic fragment by using droplet digital PCR (ddPCR) to quantify *HBB*, *HBG2*, *HBA1* and mini-LCR sequences in Berkeley mice heterozygous for the human transgenes (*n*=6). On average, there were 21.7±1.5 copies of *HBB*, 26.6±0.3 copies of *HBG2*, 9.3±0.3 copies of *HBA1* and 3.9±0.1 copies of the mini-LCR (Fig. 1D; Table S1). Based on the published sizes of each transgenic fragment, the entire transgene is calculated to be 885-1076 kb. However, this likely represents an overestimate due to the presence of incomplete transgene fragments, as indicated by different copy numbers of *HBG2* and *HBB^S^*, which were introduced within the same DNA segment (Pászty et al., 1997). Analysis of lung fibroblasts from a Berkeley mouse heterozygous for the transgene by FISH showed that the presence of a large transgene caused increased physical distance between mouse DNA probes flanking the insertion site (Fig. 1E).

### Genomic characterization of the Townes SCD mouse

**Fig. 2.**
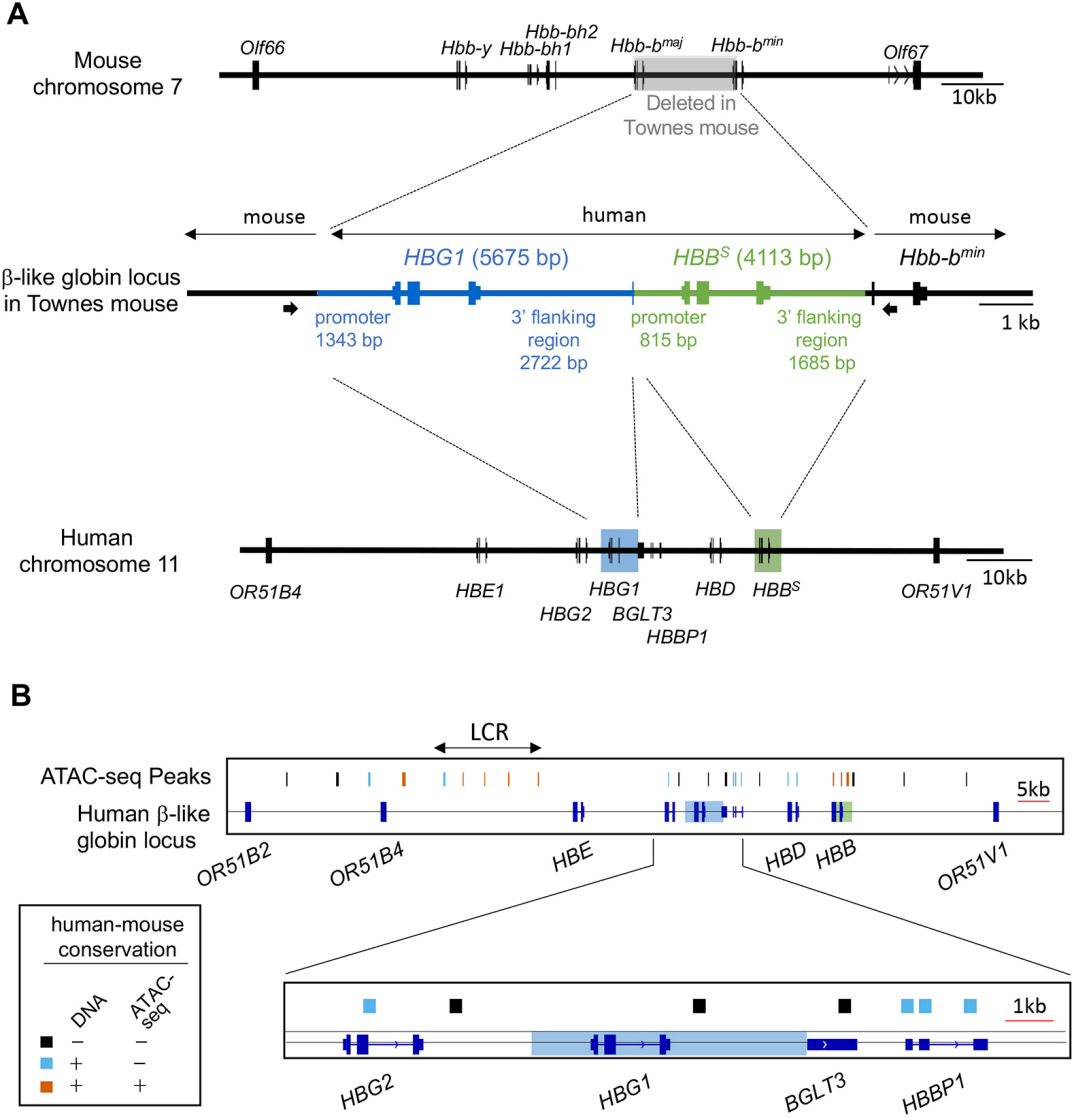
**Human globin gene structure in the Townes mouse model for SCD.** (A) The mouse (top) and human (bottom) β-like globin gene clusters are shown. In the Townes strain (middle), the adult-type mouse *Hbb-b^maj^* and *Hbb-b^min^* genes are replaced with discontinuous segments of the human fetal globin gene *HBG1* (blue) and the adult β-globin gene harboring the SCD mutation (*HBB^S^*) (green) (Wu et al., 2006). A 13.7 kb fragment containing the human globin DNAs and flanking mouse DNA (middle) was amplified by PCR using primers represented by thick arrows and analyzed by Oxford Nanopore Technologies MinION sequencing. The sizes in base pairs (bp) of transgene promoters and 3′ flanking regions are indicated. (B) Human assay of transposase-accessible chromatin sequencing (ATAC-seq) peaks in the extended human β-like globin locus (chr11:5189001-5329000, hg38 reversed) are indicated. Regions in which the underlying DNA sequence and ATAC-seq signals are conserved in the orthologous mouse locus are red. Regions with DNA sequence conservation but no ATAC-seq peaks in mice are blue. Regions with no DNA sequence conservation or ATAC-seq peaks in mice are black. Regions of *HBG1* and *HBB* present in the Townes mouse are outlined by transparent blue and green rectangles. LCR, locus control region.

We analyzed the Townes strain by performing Oxford Nanopore Technologies MinION sequencing on a 13.7 kb PCR fragment containing the human *HBG1* and *HBB* genes and flanking mouse DNA (Fig. 2A) (Jain et al., 2016). In total, 47,020 reads including 226,170,000 bases were compiled into a single 13,728 bp consensus sequence that was aligned to the human and mouse genomes (Fig. 2A; Fig. S3). The 5675 bp human γ-globin (*HBG1*) insert includes 1343 bp of promoter sequence and 2722 bp of 3′ non-coding DNA, which extends into the first 21 bp of the adjacent long non-coding RNA locus *BGLT3.* The 4113 bp *HBB^S^* insert includes 815 bp of promoter sequence and 1685 bp of non-coding 3′ DNA. These findings are consistent with the reported strategy for construction of the Townes strain (Wu et al., 2006). The human *HBB^S^* and *HBG1* transgenes include all known proximal DNA regulatory elements in the promoters and 3′ flanking regions (Antoniou et al., 1988; Doerfler et al., 2021; Martyn et al., 2017, 2018). However, we identified several human assay of transposase-accessible chromatin sequencing (ATAC-seq) signals within the extended β-like globin locus that are not conserved in mice and are not included in the Townes mouse *HBG1* or *HBB^S^* transgenes (Fig. 2B; Fig. S4). These open chromatin regions may represent human-specific *cis* elements that evolved to regulate γ-to-β-globin switching, which does not occur in mice. The absence of these elements in the Townes strain may impair expression of the *HBG1* transgene. In this regard, the *HBBP1* and *BGLT3* genes (Huang et al., 2017; Ivaldi et al., 2018) and 3′ HS1, a CTCF-binding region downstream of *HBB* (Himadewi et al., 2021), have been shown to regulate γ-globin gene expression, contain human-specific ATAC-seq peaks and are not present in the Townes strain.

### Editing of the human γ-globin gene promoters in Berkeley and Townes mice

**Fig. 3.**
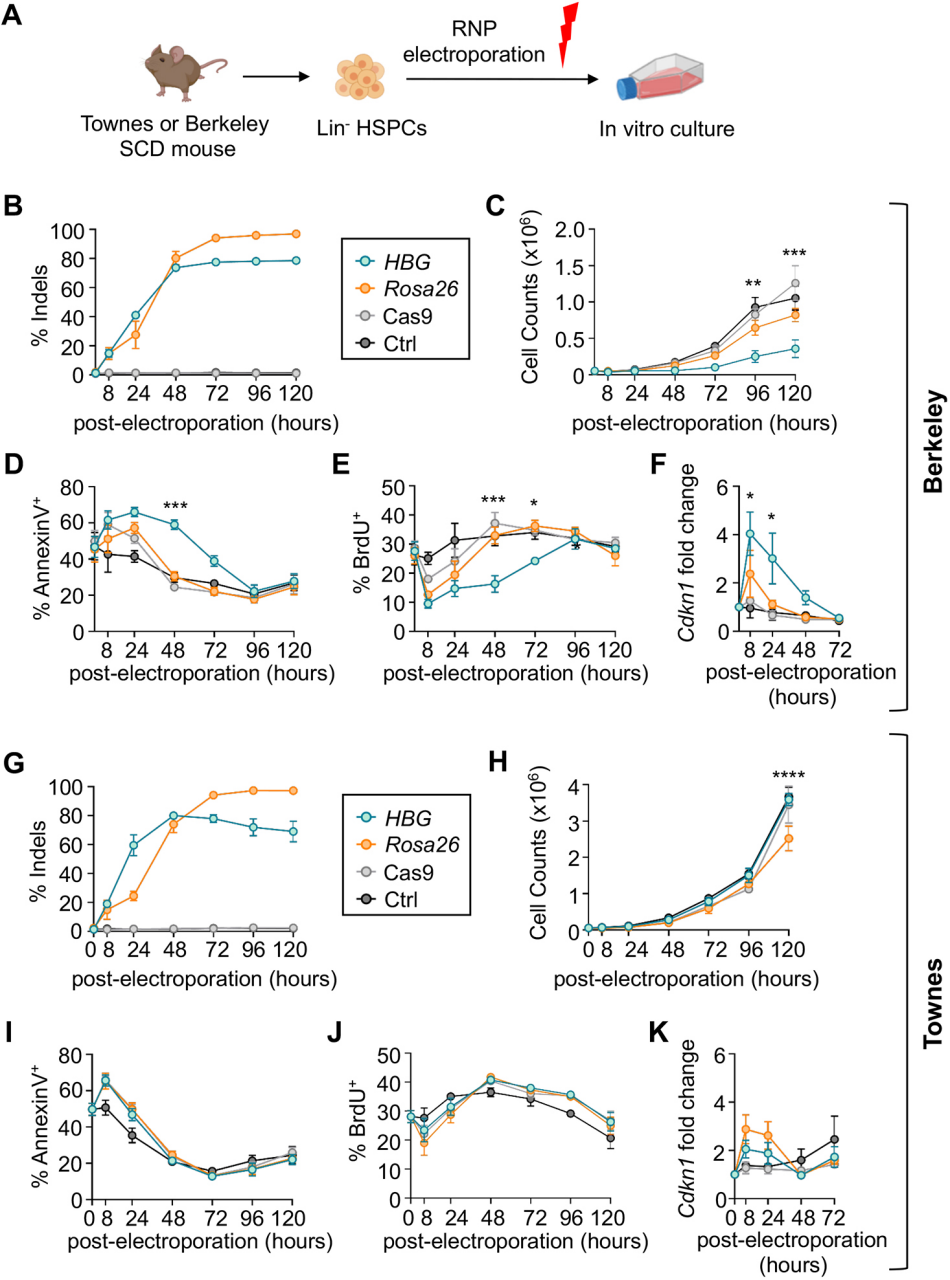
**Editing the human γ-globin transgenes impairs the viability of Berkeley but not Townes mouse hematopoietic stem and progenitor cells (HSPCs).** (A) Experimental strategy. Three million lineage-negative (Lin^−^) bone marrow cells from Berkeley (B-F) or Townes (G-K) mice were electroporated with ribonucleoprotein (RNP) complex consisting of Cas9+guide RNA (gRNA) targeting the −118 to −114 BCL11A binding site (TGACCA) in the *HBG1/2* promoter, or a control gRNA targeting *Rosa26* intron 1, and grown in culture for 5 days with mouse stem cell factor (mSCF), human FLT3 (hFLT3) ligand, mouse interleukin 3 (mIL-3) and mouse interleukin 11 (mIL-11). (B,G) On-target insertion-deletion (indel) mutations in genomic DNA, determined by next-generation sequencing (NGS) of PCR products generated across the targeted region after editing. Controls include electroporation with Cas9 alone or non-electroporated cells (Ctrl). (C,H) Live cell numbers measured by Trypan Blue exclusion. (D,I) Percentages of apoptotic (Annexin V^+^) cells measured by flow cytometry. (E,J) Percentages of S-phase cells measured by incorporation of bromodeoxyuridine (BrdU). (F,K) Fold change in the TP53-induced *Cdkn1* (p21) mRNA measured by real-time quantitative PCR (RT-qPCR), normalized to mouse *Gapdh* mRNA. All graphs show data as mean±s.e.m. from two biological replicate experiments with cells from two mice for each replicate (*n*=4 mice total). *P<0.05, **P<0.01, ***P<0.001, ****P<0.0001, by two-way ANOVA for HBG*-* versus *Rosa26*-edited cells.

Cas9-mediated disruption of a key BCL11A binding motif in the γ-globin promoters raises RBC HbF to potentially therapeutic levels (Métais et al., 2019; Traxler et al., 2016). To assess the effects of this genetic perturbation in the Berkeley and Townes mice, we isolated lineage-negative (Lin^−^) HSPCs from bone marrow, electroporated them with ribonucleoprotein (RNP) consisting of Cas9 and single-guide RNA (gRNA) targeting the BCL11A motif or the *Rosa26* locus as control, then monitored the cells in culture (Fig. 3A, Fig. S5A).

Efficient editing of Berkeley mouse HSPCs was achieved at the HBG (77.6±1.6%, *n*=4) and *Rosa26* (94.2±0.9%, *n*=4) loci by 72 h (Fig. 3B). Most edits disrupted the BCL11A binding motif (TGACCA), predicting enhanced γ-globin transcription (Fig. S5B,C). The most common edit was a 13 bp deletion found in a naturally occurring HPFH variant, which likely occurred via microhomology-mediated end joining of the Cas9-induced double-strand DNA break (DSB) (Gilman et al., 1988; Métais et al., 2019). However, HBG promoter-edited HSPCs exhibited ∼60% reduced expansion compared to controls, with increased apoptosis and cell-cycle arrest (Fig. 3C-E; Fig. S5D,E). These effects were associated with increased expression of the TP53 target gene *Cdkn1* (p21; also known as *Cdkn1a*) (Fig. 3F) (Georgakilas et al., 2017; Mauro et al., 2012). We next examined the effects of targeting the same region in Townes mice. Delivery of the *HBG1* promoter-targeting RNP to Townes mouse HSPCs resulted in efficient editing, with a similar indel pattern to that which occurred after editing Berkeley HSPCs (Fig. 3G; Fig. S5F). However, in contrast to the results obtained with Berkeley mouse HSPCs, editing of the single-copy *HBG1* promoter in Townes HSPCs produced no abnormalities in cell expansion, apoptosis, cell cycle or p21 induction compared to controls (Fig. 3H-K).

**Fig. 4.**
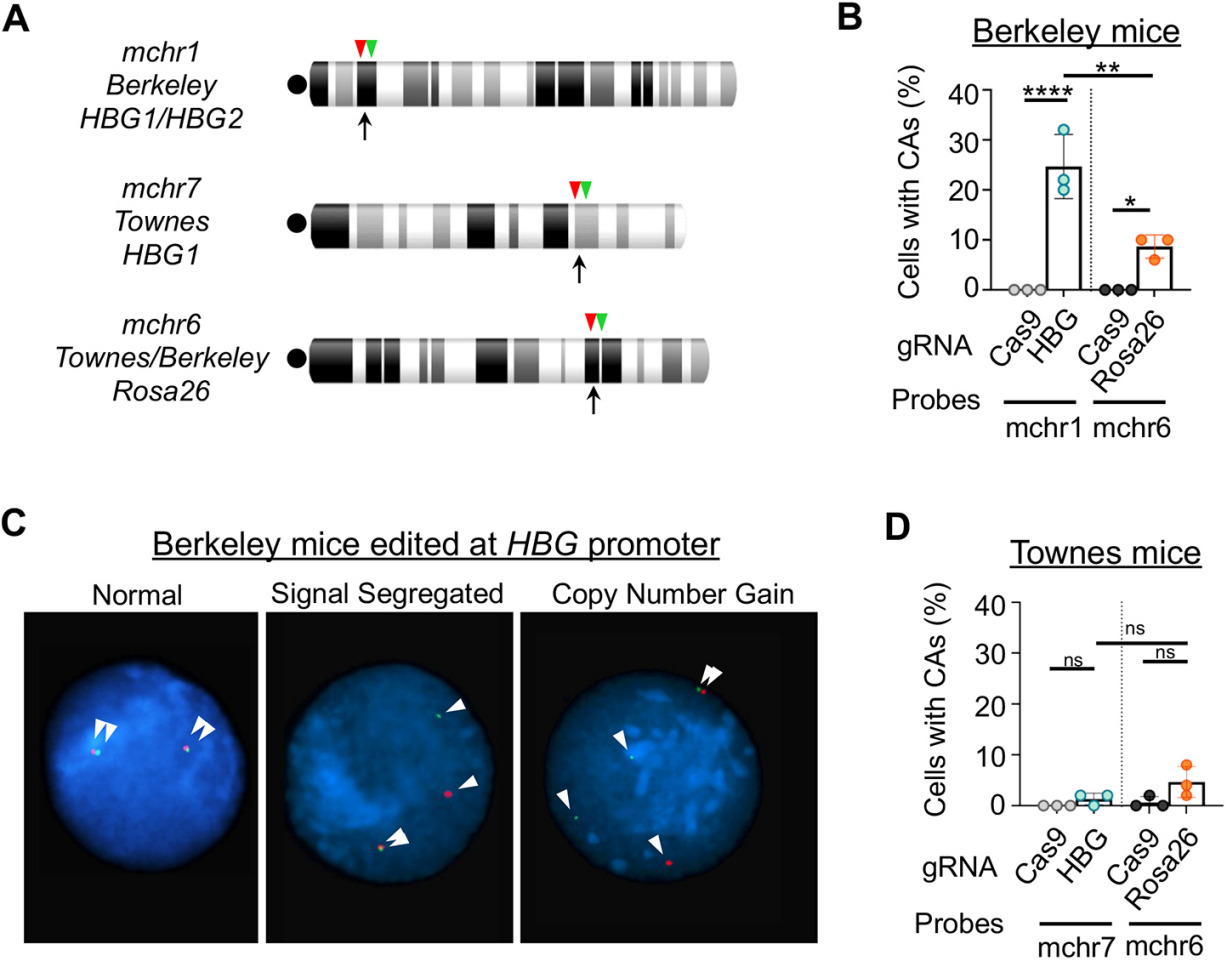
**Chromosome instability after editing the human γ-globin transgenes in Berkeley mice.** Bone marrow-derived Lin^−^ HSPCs from Berkeley or Townes mice were edited and grown for 24 h as described in Fig. 3A, then analyzed for chromosomal aberrations using FISH. (A) Chromosome ideograms showing each editing site (black arrows) and flanking centromeric (red arrowheads) and telomeric (green arrowheads) FISH probes. Centromeres are indicated by black dots. (B) Percentages of Berkeley Lin^−^ cells with chromosomal abnormalities (CAs) identified by abnormal FISH signal segregation after editing the γ-globin (HBG) promoters or *Rosa26*. (C) Representative FISH images of *HBG1/HBG2*-edited Berkeley HSPCs showing normal probe pairing, abnormal segregation of a telomeric segment or copy number gain. White arrowheads indicate probe signals. (D) Frequency of CAs after editing *HBG1* or *Rosa26* in Townes Lin^−^ HSPCs. Graphs show mean±s.e.m. of three biological replicate experiments, 50 interphase cells analyzed per sample. ns, not significant; *P<0.05, **P<0.01, ****P<0.0001, by one-way ANOVA.

Considering that the Berkeley strain harbors at least 20 copies of *HBG1*, the deleterious effects of its promoter editing likely arise from excessive DSBs, which elicit an enhanced DNA damage response that includes TP53-dependent apoptosis, cell cycle arrest and large-scale chromosomal abnormalities (Aguirre et al., 2016; Argueso et al., 2008; Heddle et al., 1991; Smith et al., 2020; Zhang et al., 2013). We edited Berkeley HSPCs at the HBG or *Rosa26* locus and performed FISH analysis 24 h later using probes that are centromeric or telomeric to the Cas9-induced DSBs (Fig. 4A). The frequency of Berkeley HSPCs with chromosomal abnormalities identified by abnormal distributions of probe signals was increased after HBG editing compared to *Rosa26* editing or electroporation with Cas9 alone (Fig. 4B,C). These abnormalities are likely underestimated due to associated reductions in cell proliferation and/or viability. In contrast, relatively few chromosomal abnormalities were observed after editing *HBG1* or *Rosa26* in Townes mouse HSPCs (Fig. 4D). Thus, a potent DNA damage response associated with a loss of cell viability occurred specifically after editing the multi-copy γ-globin transgenes in Berkeley mice.

### Editing of the HBG transgene impairs bone marrow engraftment of Berkeley HSCs

**Fig. 5.**
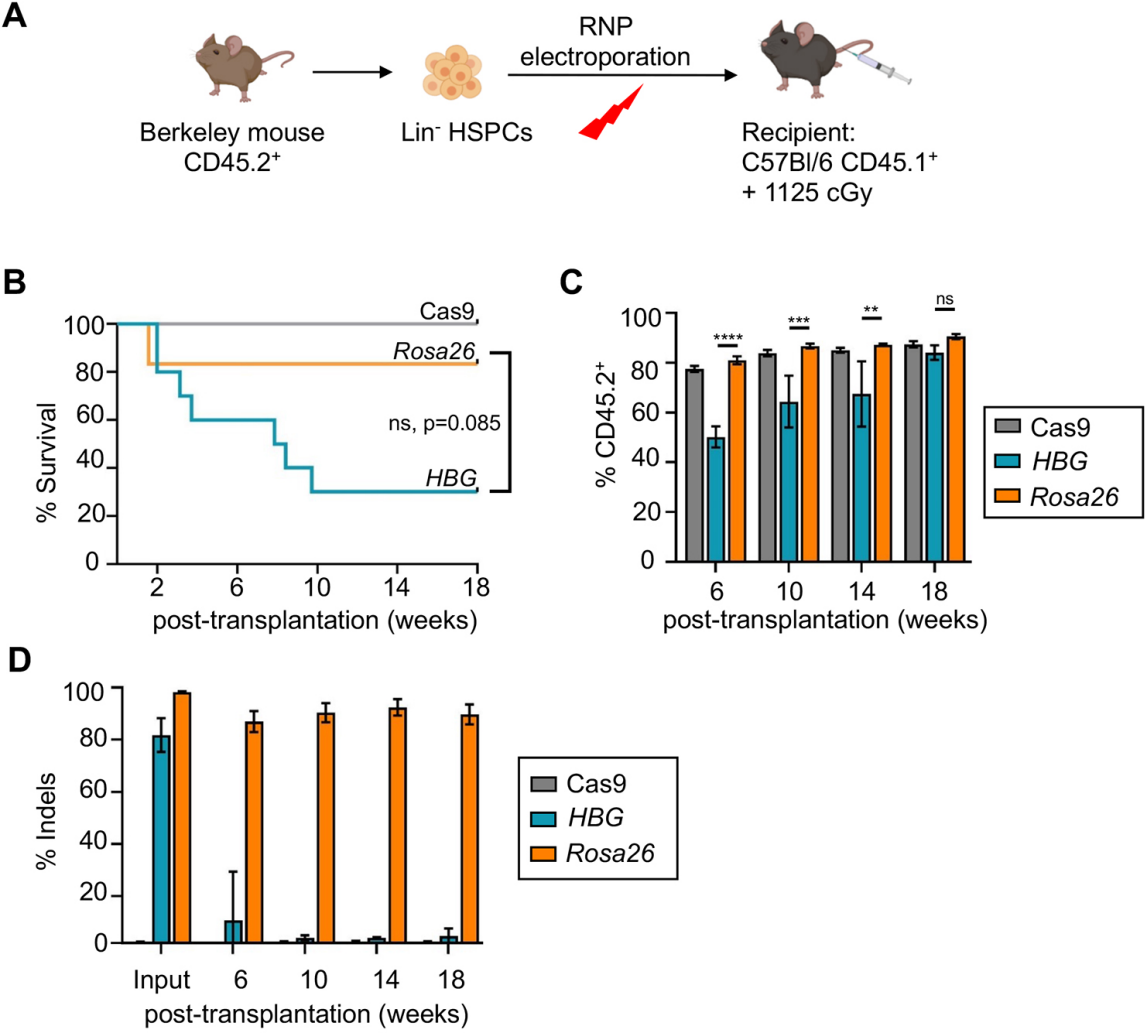
**Editing the γ-globin transgenes eliminates bone marrow engraftment of Berkeley mouse HSCs.** (A) Experimental strategy. Lin^−^ HSPCs from Berkeley mice (CD45.2) were transfected with RNP targeting *HBG1/HBG2* or *Rosa26*, transplanted into irradiated C57Bl/6 (CD45.1) hosts and analyzed serially. (B) Kaplan–Meier curves showing survival of irradiated recipients after transplantation of edited HSPCs. (C) Engraftment of *HBG1/HBG2-*edited or *Rosa26*-edited Berkeley mouse donor HSPCs over time, as determined by the fraction of circulating CD45.2^+^ mononuclear cells in recipient mice. (D) Frequency of on-target indels at the *HBG* or *Rosa26* loci in blood mononuclear cells at the indicated times following transplantation. Graphs show mean±s.e.m. of three biological replicate experiments with *n*=10 *HBG*-edited, *n*=6 *Rosa26*-edited and *n*=10 control (Cas9 only-transfected) mice. ns, not significant; **P<0.01, ***P<0.001, ****P<0.0001, by unpaired, two-tailed Student's *t*-test, survival calculations Log-rank (Mantel–Cox) test.

Lin^−^ HSPCs from Berkeley mice (CD45.2) were edited at the HBG promoter or *Rosa26* locus and injected into lethally irradiated C57Bl/6 mice (PepBoy, CD45.1) (Fig. 5A). By 18 weeks, the survival of mice transplanted with HBG-edited HSPCs (*n*=10) was 30% versus 83.3% in those transplanted with *Rosa26*-edited HSPCs (*n*=6, *P*=0.085) (Fig. 5B). The HBG-edited HSPCs exhibited delayed engraftment compared to *Rosa26*-edited HSPCs (Fig. 5C; Fig. S6). By 18 weeks, the average indel formation was 3.1±1.3% in hematopoietic cells of mice that were 95% reconstituted with HBG-edited HSPCs, compared to 89.7±3.8% indel formation in recipients of *Rosa26*-edited HSCs (Fig. 5D). Thus, editing of the multicopy HBG transgene in Berkeley HSPCs markedly impairs HSC engraftment.

### *HBG1* promoter editing in Townes HSPCs causes low-level HbF induction

**Fig. 6.**
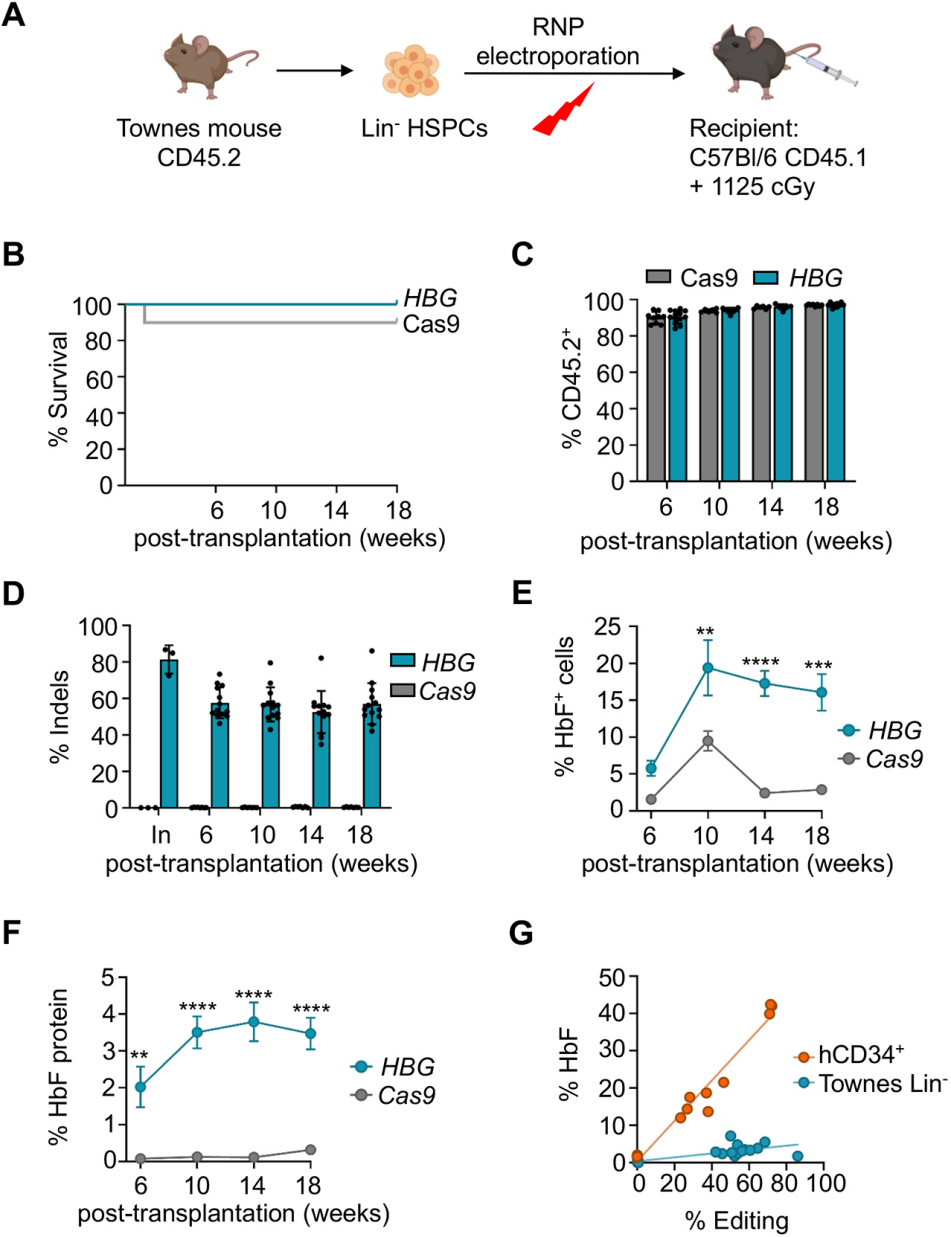
**Townes mouse HSPCs edited at *HBG1* engraft efficiently but fail to recapitulate human hereditary persistence of fetal hemoglobin (HPFH).** (A) Experimental strategy. Lin^−^ HSPCs from Townes mice were transfected with Cas9+gRNA RNP targeting the human *HBG1* promoter or Cas9 only, then transplanted into irradiated C57Bl/6 hosts. (B) Kaplan–Meier curves showing survival of recipients after transplantation of edited or control HSPCs. (C) Engraftment of donor HSPCs, as determined by the fraction of circulating CD45.2^+^ mononuclear cells in recipient mice. (D) Percentage of *HBG1* promoter indels in blood mononuclear cells after transplantation. (E) HbF immunostaining cells (F-cells) determined by flow cytometry. (F) Percentage of HbF protein in red blood cell lysates, determined by ion-exchange high-performance liquid chromatography. Graphs in C-F show data as mean±s.e.m. of three independent transplantation experiments, *n*=13 *HBG*-edited and *n*=9 Cas9 control mice. **P<0.01, ***P<0.001, ****P<0.0001, by unpaired, two-tailed Student's *t*-test. (G) %RBC HbF protein versus %peripheral blood mononuclear cell indels in recipient mice transplanted with *HBG1*-edited Townes HSPCs (teal circles) or *HBG*-edited human CD34^+^ cells from SCD patients (orange circles). Orange data points are adapted from Métais et al. (2019) with permission.

In contrast to results obtained with Berkeley mice, editing of the single-copy human *HBG1* promoter in Townes HSPCs did not impair the survival of recipients (Fig. 6A,B) or delay engraftment (Fig. 6C). At 18 weeks after xenotransplantation with *HBG1*-edited Townes HSPCs, recipients were fully engrafted, and circulating mononuclear cells harbored 57.1±3.1% on-target indels (Fig. 6D). The fraction of HbF-expressing RBCs (F-cells) measured by immuno-flow cytometry was 16.0±2.5% (*n*=13) versus 2.9±0.2% in RBCs derived from HSPCs that were transfected with Cas9 alone (*n*=9, *P*=0.0002) (Fig. 6E; Fig. S7A). The fraction of HbF in bulk lysates of RBCs derived from *HBG1* promoter-edited HSPCs was 3.5±0.4% (*n*=13), compared to 0.3±0.1% in control RBCs (*n*=9, *P*<0.0001) (Fig. 6F; Fig. S7B). At similar estimated editing frequencies, the %HbF in RBCs derived from *HBG* promoter-edited Townes HSCs was ∼7-fold less than the %HbF in the RBC progeny of human CD34^+^ cells that were edited with the same RNP, followed by transplantation into NBSGW mice (Fig. 6G) (Métais et al., 2019). The induction of HbF resulting from editing the *HBG1* promoter in the Townes mice was not sufficient to alter the levels of SCD biomarkers, including blood reticulocyte fraction, hemoglobin levels, RBC levels and spleen weight (Fig. S7C-F). Thus, disruption of the BCL11A binding motif in the *HBG1* promoter in the Townes mouse causes induction of HbF, but to levels that are markedly less than those in RBCs derived from human HSPCs edited at the same site.

## DISCUSSION

Townes and Berkeley mice are valuable for examining SCD pathophysiology (Keleku-Lukwete et al., 2015; Nasimuzzaman et al., 2019; Szczepanek et al., 2012) and the effects of various therapeutic perturbations, including drug treatment (Oksenberg et al., 2016; Shrestha et al., 2021; Tchernychev et al., 2021; Vinchi et al., 2013), lentiviral vector gene transduction (Pawliuk et al., 2001; Perumbeti et al., 2009; Pestina et al., 2009; Urbinati et al., 2018) and disruption of genes that regulate γ-to-β-globin switching, such as *Bcl11a* (Xu et al., 2011). Additionally, the Townes mouse has been useful for studying genome-editing and base-editing approaches to alter the mutant *HBB^S^* codon (Newby et al., 2021; Wilkinson et al., 2021). Despite the widespread use of these strains, the structure and regulation of their resident human globin genes is not fully defined or easily accessed through current databases or literature. This knowledge gap exists in part because the Berkeley and Townes strains were generated more than 25 and 15 years ago, respectively, when methods for genome manipulation and characterization were less advanced and the *cis*-acting DNA elements responsible for globin gene regulation were less well defined. Moreover, the Townes strain described here (Wu et al., 2006) can be mistaken for other versions of SCD mice generated by the same group (Ryan et al., 1997, 1990). The current study provides genomic characterization of ‘Townes mice’ that are available through a commercial source.

Here, we provide insights gained through efforts to establish a preclinical animal model for therapeutic induction of HbF via CRISPR/Cas9 ablation of a functionally important BCL11A binding motif in the human γ-globin promoters. Our studies provide new information on the human β-like globin sequences in the Berkeley and Townes mice, and the associated limitations in studying globin gene regulation via perturbation of genetic elements within transgenes.

The Berkeley strain was developed by creating transgenic mice via pronuclear injection of three separate human DNA segments encoding *HBA1*, *HBG2-HBG1-HBD-HBB^S^* and a ‘mini-LCR’, followed by interbreeding to eliminate the paralogous mouse genes (Pászty et al., 1997). We used ddPCR and TLA sequencing to show that the entire human transgene contains ∼22 copies of *HBB*, 27 copies of *HBG1*, nine copies of *HBA1* and four copies of the mini-LCR, inserted in random orientations into a single integration site within a gene-poor region of heterochromatin in mouse chromosome 1. The robust expression of human globin genes after integration into this region highlights the LCR as a powerful enhancer that confers high-level, integration site-independent erythroid expression of linked transgenes (Grosveld et al., 1987; Li et al., 2002). Considering that the three Berkeley transgene sequences are inserted semi-randomly and that some are likely to be fragmented, it is possible that the expression levels of different individual globin transgenes vary. Moreover, the transgene cluster may be subject to rearrangement over successive generations due to high rates of gene conversion or recombination. Efficient modification of the multicopy *HBG1/2* genes in Berkeley mouse HSPCs by CRISPR/Cas9-mediated NHEJ caused growth arrest and apoptosis accompanied by increased TP53 activity, consistent with studies showing that Cas9-induced DSBs in repetitive genomic DNA causes copy number- and TP53-dependent DNA-damage responses leading to reduced cell fitness (see Figs 3 and 5) (Haapaniemi et al., 2018; Ihry et al., 2018; Van Den Berg et al., 2018). Editing the HBG loci in Berkeley mice also led to major chromosomal rearrangements, which can occur when DSBs are present during mitosis (Heddle et al., 1991; Leibowitz et al., 2021; Zhang et al., 2013). Here, we show that editing the repetitive HBG loci in Berkeley mouse HSPCs eliminates virtually all hematopoietic stem cells. It may be possible to overcome this limitation in future preclinical studies by the use of base editors or prime editors, which are associated with lower rates of DSBs (Gaudelli et al., 2017; Komor et al., 2016).

The Townes strain was created by using homologous recombination to replace mouse α-globin genes (*Hba-a1* and *Hba-a2*) with human *HBA1* and mouse adult β-globin genes (*Hbb-b^maj^* and *Hbb-b^min^*) with separate, discontinuous segments of *HBG1* and *HBB* (or *HBB^S^*) (Wu et al., 2006). Long-range DNA sequencing of Townes mouse genomic DNA verified the exact sizes and genetic configurations of the human β-like globin loci, confirming the presence of known proximal promoter regulatory elements. Disruption of the BCL11A binding motif in the single-copy *HBG1* promoter of bone marrow-repopulating Townes mouse HSCs caused a significant induction of HbF in RBC progeny, but the magnitude was 7- to 10-fold less than what occurs after using the same Cas9-gRNA RNP to edit human HSCs (see Fig. 6E-G) (Métais et al., 2019). Interestingly, germline erythroid-specific ablation of the endogenous *Bcl11a* gene in Townes mice caused induction of HbF from <1% at baseline to ∼28% (Xu et al., 2011), contrasting with the current study showing only 3.5% HbF after disrupting the *HBG1* promoter BCL11A binding motif at an average editing efficiency of 57% (Fig. 6D,F). These findings suggest that in the Townes genomic configuration, the γ-globin promoter BCL11A binding site does not appear to be all important for repression and that other elements, including additional BCL11A binding sites, may also play a role.

Transgenic studies of mice harboring an ∼240-kb yeast artificial chromosome (YAC) encompassing the β-like globin gene cluster have shown that the order of human β-like globin genes and their DNA context influence developmental patterns of expression (Gaensler et al., 1993; Kollias et al., 1986; Peterson et al., 1993; Strouboulis et al., 1992). In contrast to our findings in the Townes strain, disruption of the γ-globin promoter BCL11A motif in HSCs from mice harboring the β-globin YAC resulted in a stronger β-to-γ-globin shift in erythroid progeny, comparable to the effects seen in RBCs after Cas9 disruption of the same motif in human HSCs (Li et al., 2018; Traxler et al., 2016). Disruption of the same BCL11A binding motif by installing the *HBG1* HPFH variant −117 G>A in β-globin YAC transgenic mice also caused a robust induction of γ-globin (Lin et al., 2000; Peterson et al., 1995). These data indicate that the Townes strain lacks key *cis*-regulatory elements for controlling human β-globin switching, which could include the long non-coding RNA locus, *BGLT3*, the globin pseudogene *HBBP1* and the 3′ HS1 downstream of *HBB* (Fig. 2B) (Himadewi et al., 2021; Huang et al., 2017; Ivaldi et al., 2018). Efforts to model SCD in mice harboring the β-globin YAC have been complicated by excessive perinatal death, most likely because the human γ-to-β-globin gene switch occurs during mid-gestation in mice, thereby eliminating the protective effects of HbF expression during birth-associated hypoxic trauma (Chang et al., 1998).

In summary, this study provides improved physical and functional characterization of the human globin transgenes in the Berkeley and Townes models for SCD and illustrates the limitation of these strains for evaluating therapeutic genome editing of autologous HSPCs to induce RBC HbF. Specifically, HSPCs from Berkeley mice do not survive Cas9 genome editing of the repetitive human HBG loci, and Townes mice, which harbor limited segments of *HBG1* and *HBB^S^*, do not recapitulate the effects of a human HPFH-like mutation, in part because key DNA-regulatory elements are missing. Our findings better define the genomic structures of these mouse models and illustrate how multiple *cis*-regulatory elements contribute to the complex developmental regulation of β-like globin gene expression.

## MATERIALS AND METHODS

### Mice

Berkeley mice (The Jackson Laboratory, Stock #003342) and Townes mice (The Jackson Laboratory, Stock #013071) were maintained in the St. Jude Children's Research Hospital (St. Jude) Animal Resource Center. Genotyping and breeding were performed as per suggested protocols by The Jackson Laboratory. Mice were housed and handled according to recommendations of the Guide for the Care and Use of Laboratory Animals from the National Institutes of Health (NIH). All animal experiments were approved by the St. Jude Institutional Animal Care and Use Committee (IACUC).

### TLA sequencing of the Berkeley mouse transgene

Spleens from 8-week-old mice were homogenized, passed through a 40 µm filter and rinsed with phosphate buffered saline (PBS)+10% fetal bovine serum (FBS). Erythrocytes were lysed, and the remaining mononuclear cells were cryopreserved in PBS with 10% dimethyl sulfoxide and 10% fetal calf serum. To perform TLA, cells were treated with formaldehyde to cross-link DNA segments in close physical proximity, digested with NlaIII, treated with DNA ligase, de-crosslinked, digested with Nsp1 to form ∼2 kb fragments and circularized. The DNA was then amplified with inverse PCR primer pairs complementary to each of the three transgenic fragments (Table S2) and analyzed by next-generation DNA sequencing.

### Berkeley mice sequence coverage analysis

Paired-end reads from TLA sequencing were aligned to a hybrid reference sequence of the mm9 genome combined with the human transgene loci using BWA (v 0.7.16a-r1181) (Li, 2011). BedGraph files were generated with genomeCoverageBed from bedtools (Quinlan and Hall, 2010). Bigwig files used for read coverage images were generated with bedGraphToBigWig (Kent et al., 2010).

### FISH analysis

FISH was performed by the St. Jude Cytogenetics core facility. Lin^−^ cells were incubated with colcemid for 4 h then harvested by routine cytogenetic methods. For FISH analysis, BAC clones were purchased from BACPAC Resources (bacpacresources.org; Children's Hospital Oakland Research Institute, Oakland, CA, USA), labeled with either red-dUTP (AF594, Molecular Probes) or green-dUTP (AF488, Molecular Probes), and used as hybridization probes in signal segregation studies (Table S3). Labeled probe pairs were combined with 100 ng/ml sheared mouse DNA and hybridized to interphase and metaphase cells in 50% formamide, 10% dextran sulfate and 2× saline sodium citrate (SSC) at 37°C for 16 h. Cell nuclei were stained with 2.5 mg/ml 4′,6-diamidino-2-phenylindole (DAPI), imaged using a Nikon E800 microscope (Nikon PlanApo 60×/1.40 NA oil objective), Nikon Nis Elements software and a Hamamatsu Orca 4.0 camera.

### ddPCR for copy number evaluation

Genomic DNA from blood was extracted from six Berkeley mice heterozygous for the human β-like globin transgenes using a Qiagen DNeasy kit following the manufacturer's guidelines. DNA was digested with CviQI (NEB) and was used as the template for PCR. Primer-probe sets and ddPCR Supermix for Probes (no dUTP) were purchased from Bio-Rad. Droplet generation and analysis was performed using an QX200 AutoDG ddPCR system and QuantaSoft software (Bio-Rad). The following ready-made and custom primer-probe sets were used to amplify the Berkley transgene and mouse *Fzd2* for normalization: *HBB* (assay ID: dHsaCNS862004688), *HBA1* (assay ID: dHsaCNS896874868), *HBG2* (assay ID: dHsaCNS599713829), miniLCR (assay ID: dHsaCNS153738777) and *Fzd2* (assay ID: dMmuCNS863358151).

### Oxford Nanopore Technologies MinION sequencing of the *HBG1*-*HBB* DNA insert in Townes mice

Oxford Nanopore Technologies MinION sequencing was performed on a 13,728 bp genomic DNA fragment generated from peripheral blood mononuclear cells by PCR using primers complementary to mouse DNA spanning the human *HBG1*-*HBB* DNA insert (Table S2). Amplification was performed using PrimeSTAR GXL DNA Polymerase (Takara Bio, R050A) under two-step PCR conditions (98°C for 10 s, 68°C for 16 min repeated for 30 cycles), followed by electrophoresis on a 1% agarose gel and fragment purification using a QIAQuick Gel Extraction kit (Qiagen, 28704). A library was prepared using a Ligation Sequencing Kit (Oxford Nanopore Technologies, SQK-LSK109) according to the manufacturer's instructions (Lambda Control Experiment protocol SQK-LSK109 available at https://community.nanoporetech.com/docs/prepare/library_prep_protocols/lambdacontrol-sqk-lsk109/v/cde_9062_v109_revag_14aug2019). The library was loaded onto a MinION flow cell (R9.4.1, Oxford Nanopore Technologies, FLO-MIN106D) and sequenced for 1 h. Reads were analyzed using MinKnow software (Loose et al., 2016).

### Townes consensus sequence

Reads determined from MinKnow software following the MinION run, were aligned to mouse β-globin DNA and the human *HBB* and *HBG1* genes (Loose et al., 2016). The genomic sequence for the mouse 129S1/SvImJ was used as the similarity of matching reads was higher than the mm10 mouse reference strain (C57BL/6J). A hybrid reference sequence was generated from the aligned mouse and human sequences as well as unaligned sequence. A consensus sequence was generated from the BAM alignment files to the hybrid reference sequence using samtools pileup (Li, 2011).

### ATAC-seq tracks

Processed human erythroid-specific ATAC-seq peaks were retrieved from Gene Expression Omnibus (GEO; accession GSE115672). (Ludwig et al., 2019) Peak locations were mapped to the extended mouse β-globin region using pslMap (Zhu et al., 2007), and a matching result was used to classify the peak as conserved. If the matching mouse sequence overlapped mouse erythroid ATAC-seq peaks (GEO accession GSM4255752), the human peaks were defined as overlapping mouse ATAC-seq peaks.

Erythroid ATAC-seq data from a single donor (SRX4197882 to SRX4197887) (Ludwig et al., 2019) were downloaded from the Sequence Read Archive (SRA) database. Reads were trimmed using skewer (Jiang et al., 2014) and then mapped to the hg38 genome using BWA mem (Li and Durbin, 2010). BigWig tracks were generated with the bamCoverage (v3.2.0) function from Deeptools (Ramirez et al., 2016). The mouse ATAC-seq signal tracks were retrieved from GEO (GSM4255752).

### Lin^−^ cell purification and culture

Bone marrow from 8- to 14-week-old male and female mice was flushed from femurs, tibias, hip bones and humeri. Lin^−^ cells were enriched from bone marrow by RBC lysis (ACK Lysing Buffer, Quality Biological, 118-156-101) followed by negative immunoselection using a mouse Lineage Cell Depletion Kit (Miltenyi, 130-090-858). The Lin^−^ cells were maintained in StemSpan serum-free expansion medium (SFEM) supplemented with mouse stem cell factor (mSCF; 100 ng/ml), mouse interleukin 3 (mIL-3; 10 ng/ml), mouse interleukin 11 (mIL-11; 100 ng/ml), human FLT3 (hFLT3) ligand (100 ng/ml) and penicillin–streptomycin (PenStrep; 1×) for 24 h before electroporation with Cas9-gRNA RNP. Viable cells were enumerated in PBS with 10% Trypan Blue using a Countess II (Invitrogen).

### Cas9 genome editing of Lin^−^ cells

Chemically modified single gRNAs were obtained from Synthego (*Rosa26*, ACTCCAGTCTTTCTAGAAGA) or Trilink (*HBG1/2* promoter, CTTGTCAAGGCTATTGGTCA). RNPs were generated in HF150 electroporation buffer (10 mM HEPES, 150 mM NaCl) by mixing Cas9 protein (Berkeley Macrolabs) and gRNA at either a 1:2 (*HBG1/2*) or 1:3 molar (*Rosa26*) ratio and incubating for 30 min at room temperature. For editing, 3-5×10^6^ cells were resuspended in 50 µl Neon Buffer T and mixed with RNP (40 nM Cas9). Electroporation was performed using a ThermoFisher Scientific Neon Transfection system (program 5: 1700 pulse voltage, 20 pulse width, one pulse) in 100 µl tips and electroporation Buffer E2.

### NGS analysis

DNA for NGS was purified (>100,000) using a DNeasy Blood & Tissue Kit (Qiagen, 69504). Alternatively, small numbers of cells (<100,000) were resuspended in lysis buffer (100 mM Tris-HCl pH 8.0, 5 mM EDTA, 1% SDS), heated (50°C for 1 h, 85°C for 30 min) and used directly in subsequent NGS reactions. Targeted amplicons were generated using gene-specific primers with partial Illumina adapter overhangs (Table S2) and analyzed by NGS (Sentmanat et al., 2018). Amplicons were indexed in a second PCR and pooled with other targeted amplicons for other loci to create sequence diversity. Additionally, 10% PhiX Sequencing Control V3 (Illumina) was added to the pooled amplicon library prior to running the sample on a Miseq Sequencer System (Illumina) to generate paired 2×250 bp reads. Samples were demultiplexed using the index sequences, fastq files were generated, and NGS analysis was performed using CRIS.py (Connelly and Pruett-Miller, 2019).

### Gene expression analysis

RNA from ∼200,000 cells was extracted with an RNeasy Plus Mini Kit (Invitrogen, 74134), and reverse transcribed using an iScript cDNA Synthesis Kit (Bio-Rad, 1708890). *Cdkn1* expression was quantified by SYBR Green qPCR (Power SYBR Green PCR Master Mix, ThermoFisher Scientific, 4368577) using a QuantStudio 6 Real-Time PCR System (ThermoFisher Scientific), normalized to *Gapdh* mRNA. Primer sequences are listed in Table S2.

### Hematopoietic stem cell transplantation

Following electroporation with RNP for genome editing, Lin^−^ cells were incubated overnight in StemSpan SFEM with mSCF, mIL-3, mIL-11 and hFLT3 ligand at the concentrations noted above, then either maintained in culture for subsequent indel analysis, or transplanted into lethally irradiated (1125 cGy) 8- to 12-week-old female PepBoy recipients (CD45.1, The Jackson Laboratory, Stock #002014). For transplantation, 1×10^6^ cells were resuspended in 200 µl PBS and injected into the tail vein of recipients. Donor engraftment was determined by flow cytometry to detect differences in CD45 alleles between donor and recipient.

### Peripheral blood and histological analyses

Peripheral blood of recipient mice was analyzed serially between 0 and 18 weeks after transplantation. Blood was collected retro-orbitally using heparinized micro-hematocrit capillary tubes (Fisher Scientific, 22-362-566). Complete blood counts were determined on a FORCYTE Veterinary Hematology Analyzer. Blood smears were performed using modified Romanowsky methanolic staining and Eosin and thiazin methods. Tissue histology was determined by standard methods.

### Flow cytometry assays

Apoptotic cells were detected by flow cytometry using propidium iodide (1:20 dilution) and anti-Annexin V-FITC (1:20 dilution; FITC Annexin V Apoptosis Kit, BD Biosciences, 556547). Cell cycle analysis was performed by incubating cells with bromodeoxyuridine (BrdU; 1 mM) for 1 h and analyzed by flow cytometry for 7AAD (1:20 dilution) and anti-BrdU-FITC (1:50 dilution) using a FITC BrdU Flow Kit (BD Pharmingen, 559619). For determination of F-cells, 0.5 µl of peripheral blood was fixed with 0.05% glutaraldehyde, permeabilized with 0.1% Triton X-100 and stained with anti-HbF-APC (1:20 dilution; Clone HBF-1, Invitrogen, MHFH05). To determine donor engraftment, 5-10 ml of peripheral blood was incubated in RBC lysis buffer, washed with PBS (0.1% bovine serum albumin) and stained with mouse anti-CD45.1-PE (1:50 dilution; Clone A20, BD Pharmingen, 553776) and mouse anti-CD45.2-FITC (1:50 dilution; Clone 104, BD Pharmingen, 561874). To determine reticulocyte fraction, 1 µl of peripheral blood was stained in 1 ml BD Retic-COUNT (BD Biosciences, 349204), according to the manufacturer's instructions.

### High-performance liquid chromatography (HPLC)

Ion-exchange HPLC was performed using a Prominence HPLC System (Shimadzu Corporation). RBCs from 1 µl whole blood were lysed in 200 µl Hemolysate Reagent (0.005 M EDTA, 0.07% KCN; Helena Laboratories, 5125). Approximately 3-5 µl was fractionated on a PolyCatA column (Analytical Instruments PolyLC, 202CT0510). Proteins were quantified by light absorbance at 220 nm, 280 nm and 418 nm using a diode array detector. %HbF was determined as the area of the HbF peak/sum divided by the area of all Hb tetramer peaks [%HbF=HbF/(HbS+HbF)].

### Statistical methods

Statistical analysis was performed using GraphPad Prism. For comparisons in transplantation experiments, statistics comparing each time point were calculated by unpaired, two-tailed Student’s *t*-test. Data from all other experiments were analyzed using one- or two-way ANOVA multiple comparisons.

## Supplementary Material

Supplementary information
