# Supplementary material for: Limitations of mouse models for sickle cell disease conferred by their human globin transgene configurations
Source: Dis Model Mech. 2022 Jul 7;15(6):dmm049463. doi: 10.1242/dmm.049463 (PMC9277148; doi:10.1242/dmm.049463)
Supplement: Supplementary information [file dmm-15-049463-s1.pdf]

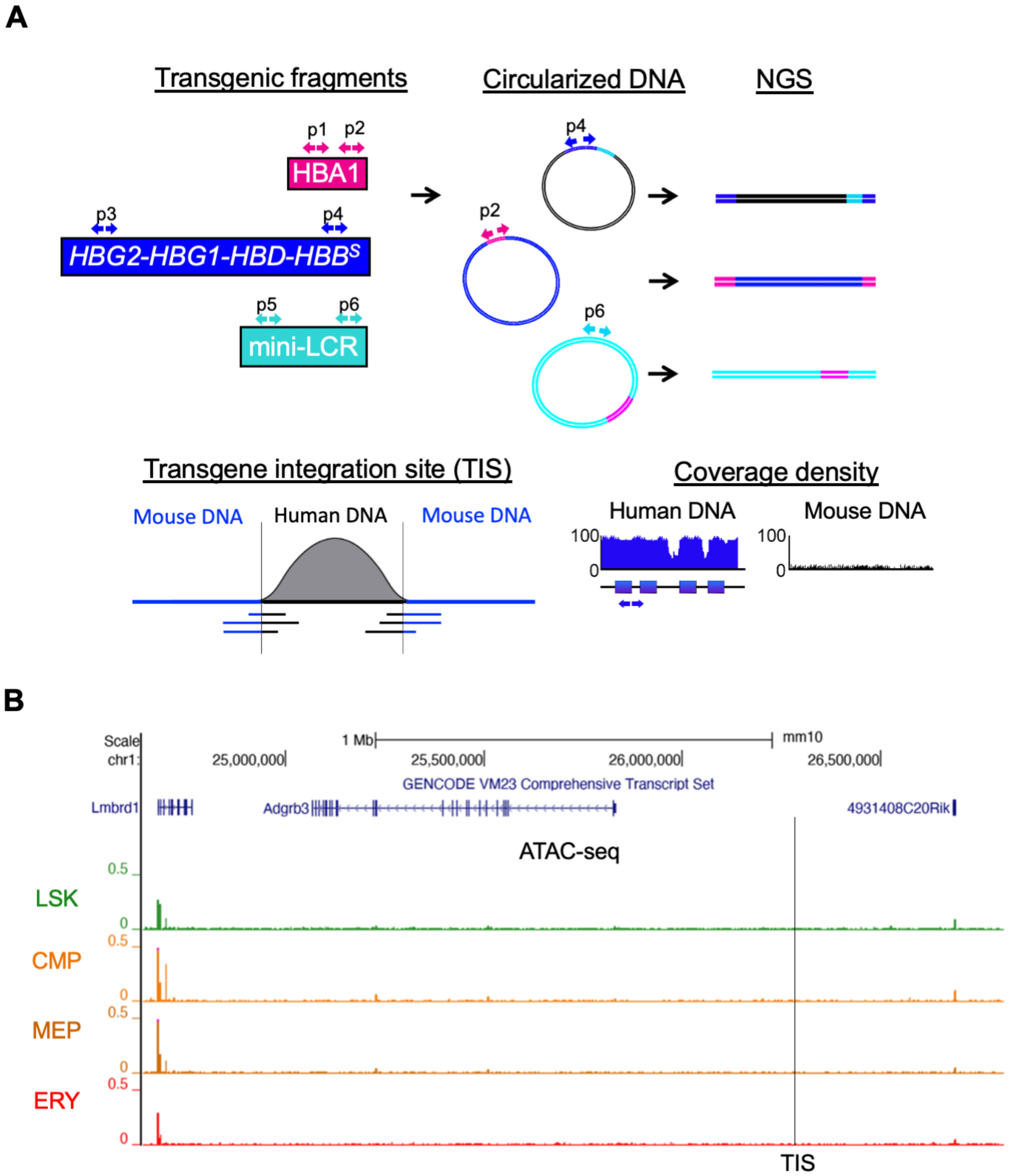

Fig. S1 (related to Figure 1). Analysis of the Berkeley mouse transgene. A) Target locus amplification (TLA) sequencing to detect the 3 Berkeley human transgenes and mouse DNA junctions. The TLA protocol to generate DNA circles containing genomic sequences in physical proximity within cells is described in Methods. Two outward-facing primer sets, designated p1-p6, were used to selectively amplify DNA from each transgene within the DNA circles, then sequenced by NGS. The transgene integration site (TIS) was determined by identifying DNA sequence reads that align to the human and mouse genomes. We identified multiple DNA fusion reads linking different transgenic fragments to each other or to mouse DNA at a single TIS. Coverage density was determined by aligning NGS reads to mouse or human genomes. **B)** ATAC-seq data from the BX VISION Browser showing nuclease accessible regions near the TIS in mouse hematopoietic cell lineages.

Disease Models & Mechanisms • Supplementary information

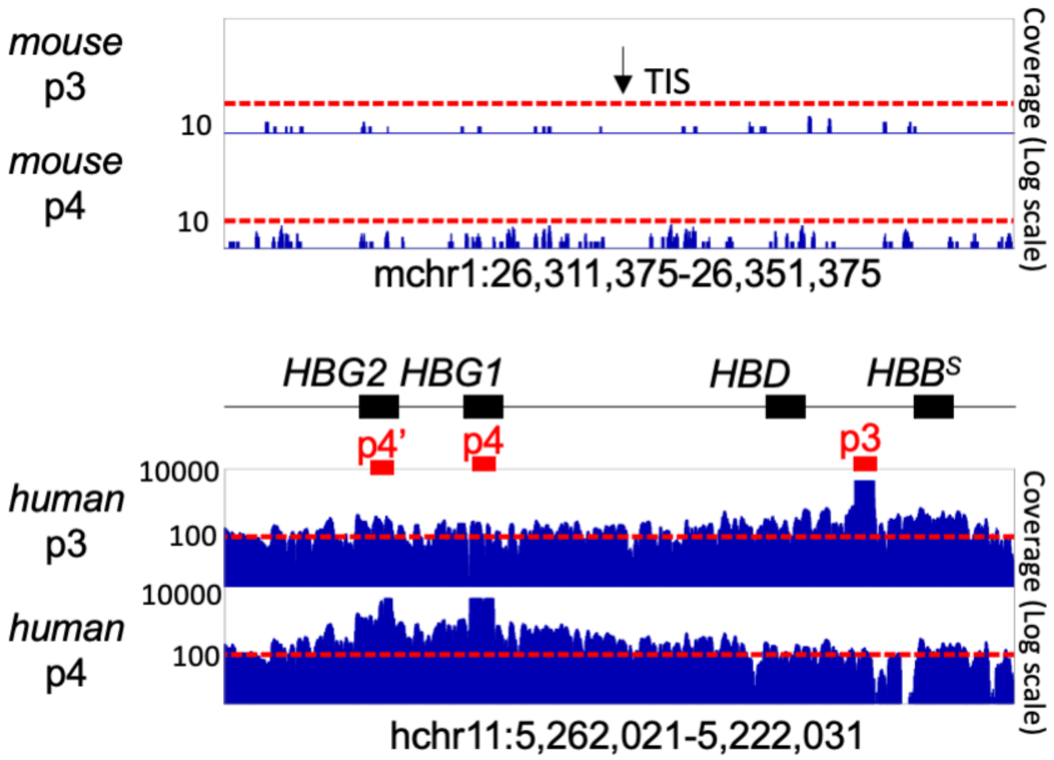

Fig. S2 (related to Figure 1). Sequence coverage (log scale) of NGS data derived from TLA primer sets 3 and 4 (Figure S1 and Table S1) aligned to the mouse and human genomes. Red dotted line indicates median coverage across region. The TIS is indicated by a black arrow in the mouse genome. Regions amplified by each primer set are indicated by red rectangles, p4' is an additional binding site for p4 based on sequence identity within the duplicated *HBG2* and *HBG1* genes.

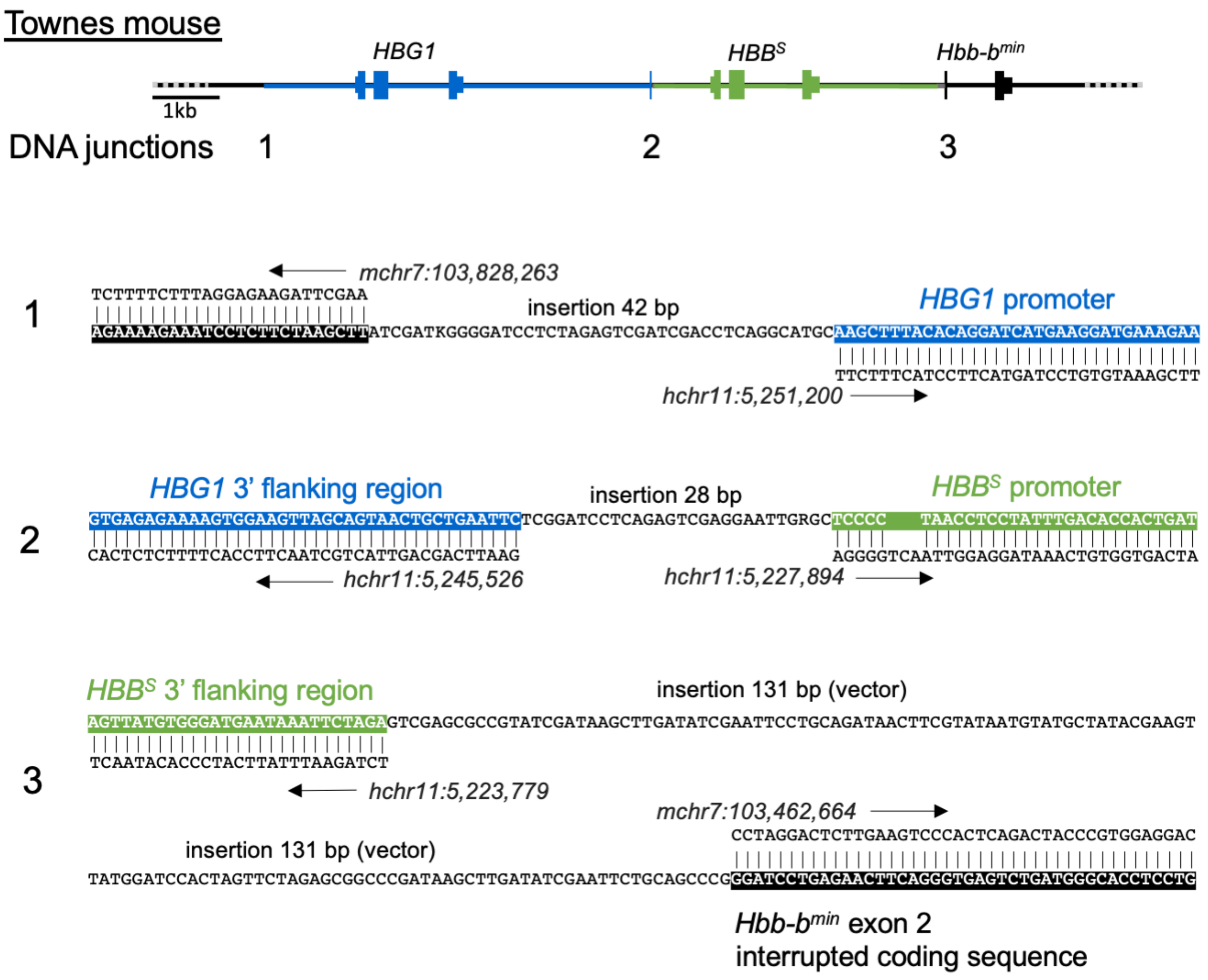

Fig. S3 (related to Figure 2). The mouse-human and human-human DNA junctions in the Townes strain. The diagram on top shows a map of the human *HBG1* (blue) and *HBB* (green) genes embedded in the mouse β-like globin locus (black). Three DNA fusions are indicated in the diagram, with the corresponding sequences aligned to consensus mouse or human genomic sequences shown below. The genome coordinates located at each breakpoint are indicated.

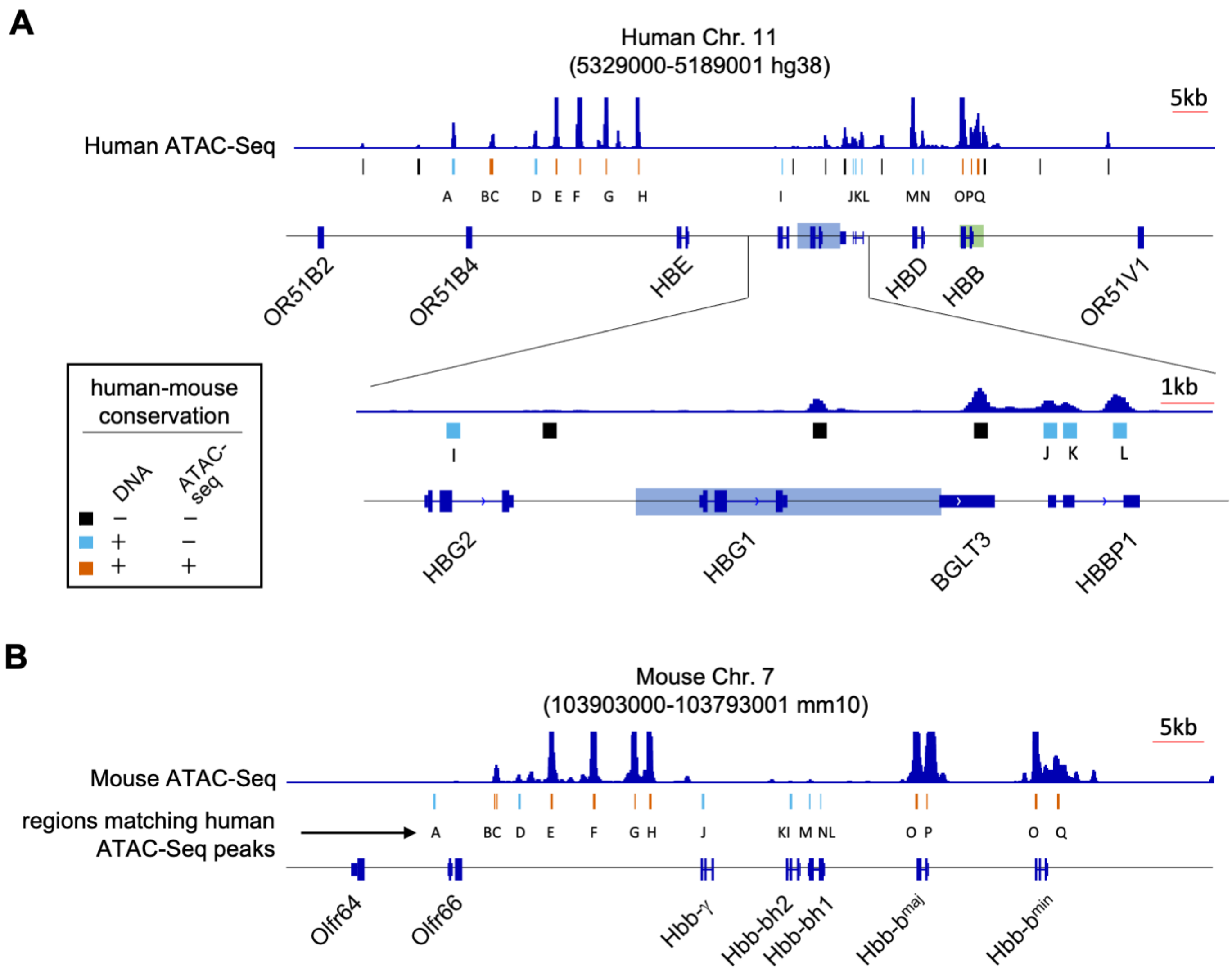

Fig. S4 (related to Figure 2). ATAC-seq peaks in the human and mouse β-like globin loci. A) Human ATAC-seq peaks in the extended human β-like globin locus (chr11:5189001-5329000, hg38 reversed) are indicated. Regions in which the underlying DNA sequence and ATAC-seq signals are conserved in the orthologous mouse locus are red. Regions with DNA sequence conservation but no ATAC-seq peaks in mice are blue. Regions with no DNA sequence conservation or ATAC-seq peaks in mice are black. Blue or red ATAC-seq peaks are labeled A to Q. Regions of *HBG1* and *HBB* present in the Townes mouse are outlined by transparent blue and green rectangles. **B)** The mouse extended β-like globin locus (mchr7:103783001-103903000, mm10 reversed) showing ATAC-seq signals across regions with sequence similarity to human DNA regions A to Q shown in panel A.

A

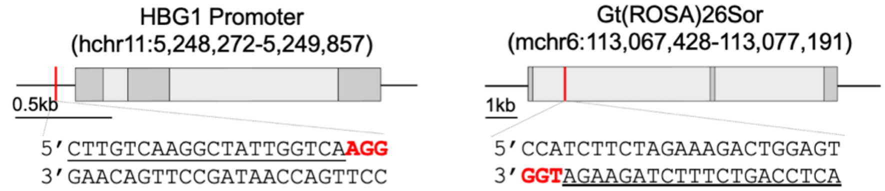

B

HBG Indel Profile - Berkeley

GGCCAGCCTTGCCTTGACCAATAGCCTTGACAAGGCAAACTTGACCAATAG	Indel	Frequency
GGCCAGCCTTGCCTTGA-------------CAAGGCAAACTTGACCAATAG	-13	31.9
GGCCAGCCTTGCCTTGACCAATAGCCTTGACAAGGCAAACTTGACCAATAG	0	23.4
GGCCAGCCTTGCCTTGA---ATAGCCTTGACAAGGCAAACTTGACCAATAG	-3	6.4
GGCCAGCCTTGCCTTGA--AATAGCCTTGACAAGGCAAACTTGACCAATAG	-2	6.1
GGCCAGCCTTGCCTTGAC-AATAGCCTTGACAAGGCAAACTTGACCAATAG	-1	5.2
GGCCAGCCTTGCCTTGA----TAGCCTTGACAAGGCAAACTTGACCAATAG	-4	3.9
GGCCAGCCTTGCCTTGAC--------TTGACAAGGCAAACTTGACCAATAG	-8	3.0
GGCCAGCCTTG------------------ACAAGGCAAACTTGACCAATAG	-18	2.4
		76.6

C

Rosa26 Indel Profile – Berkeley

GATCTGCAACTCCAGTCTTTCTAGAAGATGGGCGGGAGTCTTCTGGGCAGGCTTAAAG	Indel	Frequency
GATCTGCAACTCCAGTCTTTCTAGA-GATGGGCGGGAGTCTTCTGGGCAGGCTTAAAG	-1	19.5
GATCTGCAACTCCAGTCTTTCTAGA---TGGGCGGGAGTCTTCTGGGCAGGCTTAAAG	-3	17.4
GATCTGCAACTCCAGTCTT-----------------------CTGGGCAGGCTTAAAG	-23	15.6
GATCTGCAACTCCAGTCTTTCTAGAAGATGGGCGGGAGTCTTCTGGGCAGGCTTAAAG	0	6.1
GATCTGCAACTCCAGTCTTTCT----------------------GGGCAGGCTTAAAG	-22	3.5
GATCTG---------------------------------------GGCAGGCTTAAAG	-39	3.3
GATCTGCAACT-------------------GGCGGGAGTCTTCTGGGCAGGCTTAAAG	-19	3.0
		93.9

D

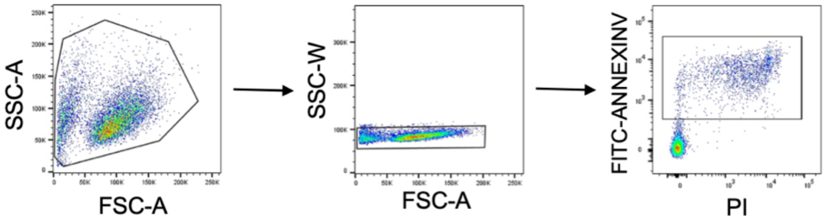

E

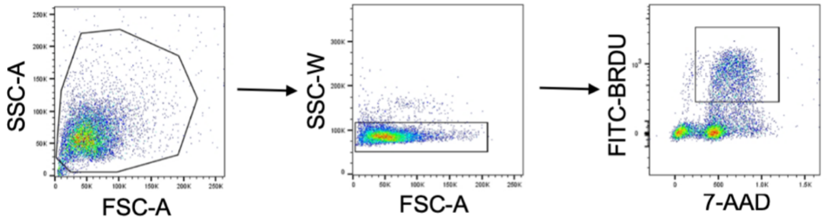

F

HBG Indel Profile - Townes

GGCCAGCCTTGCCTTGACCAATAGCCTTGACAAGGCAAACTTGACCAATAG	Indel	Frequency
GGCCAGCCTTGCCTTGAC-------------AAGGCAAACTTGACCAATAG	-13	27.7
GGCCAGCCTTGCCTTGACCAATAGCCTTGACAAGGCAAACTTGACCAATAG	0	22.0
GGCCAGCCTTGCCTTGA-CAATAGCCTTGACAAGGCAAACTTGACCAATAG	-1	8.7
GGCCAGCCTTG------------------ACAAGGCAAACTTGACCAATAG	-18	5.2
GGCCAGCCTTGCCTTGA---ATAGCCTTGACAAGGCAAACTTGACCAATAG	-3	4.2
GGCCAGCCTTGCCTTGA--AATAGCCTTGACAAGGCAAACTTGACCAATAG	-2	3.1
GGCCAGCCTTGC------CAATAGCCTTGACAAGGCAAACTTGACCAATAG	-6	2.8
GGCCAGCCTTGCCTTGA----TAGCCTTGACAAGGCAAACTTGACCAATAG	-4	2.4
		76.1

Disease Models & Mechanisms • Supplementary information

Fig. S5 (related to Figure 3) Cas9 editing of the γ-globin genes in Berkeley or Townes mice. Lineage negative (Lin$^-$) cells from bone marrow were edited by electroporation with Cas9-sgRNA ribonucleoprotein (RNP), followed by incubation in StemSpan SFEM supplemented with mouse stem cell factor (mSCF, 100 ng/ml), mouse interleukin 3 (mIL-3, 10 ng/ml), mouse interleukin 11 (mIL-11, 100 ng/ml), human FLT3 ligand (hFLT3 ligand, 100 ng/ml) and penicillin-streptomycin (PenStrep, 1X). **(A)** Sequences of single guide RNAs (sgRNAs) targeting the *HBG* promoter BCL11A binding site or *Rosa26* intron 1; PAM sequences are indicated in red. **(B,C**) High-frequency (>2%) insertion-deletion (indel) mutations detected by NGS at 72 hours after editing Lin- bone marrow cells from Berkeley mice. **(D)** Gating strategy for Annexin V flow cytometry to assess apoptosis after editing. **(E)** Gating strategy for flow cytometry to measure BrdU uptake after editing. **(F)** Indel profiles at the *HBG* promoter at 72 hours after editing Lin$^-$ bone marrow cells from Townes mice.

Disease Models & Mechanisms • Supplementary information

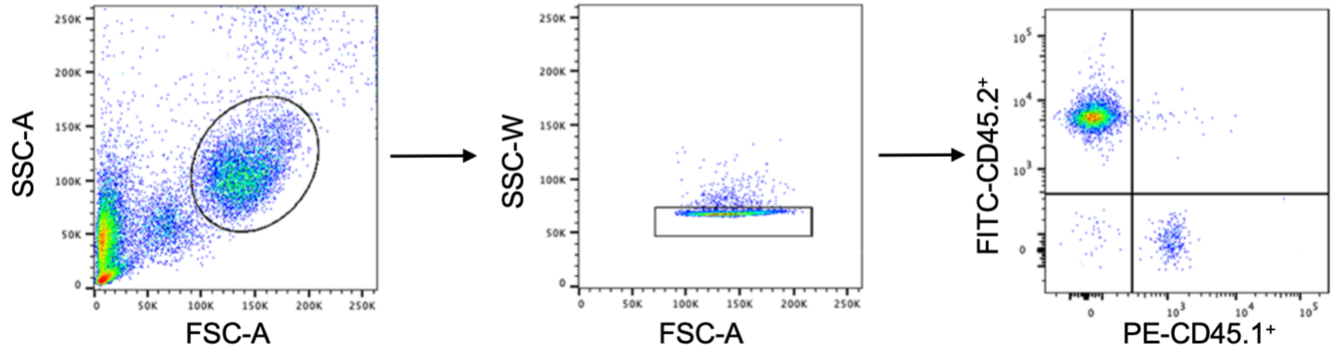

Fig. S6 (related to Figure 5). Engraftment of donor cells after mouse-to-mouse hematopoietic stem cell transplantation (HSCT). Flow cytometry gating strategy to quantify the fraction of circulating donor-derived CD45.2$^+$ mononuclear cells following HSCT int lethally irradiated CD45.1 recipient mice. Engraftment is calculated as %CD45.2/(%CD45.1+%CD45.2).

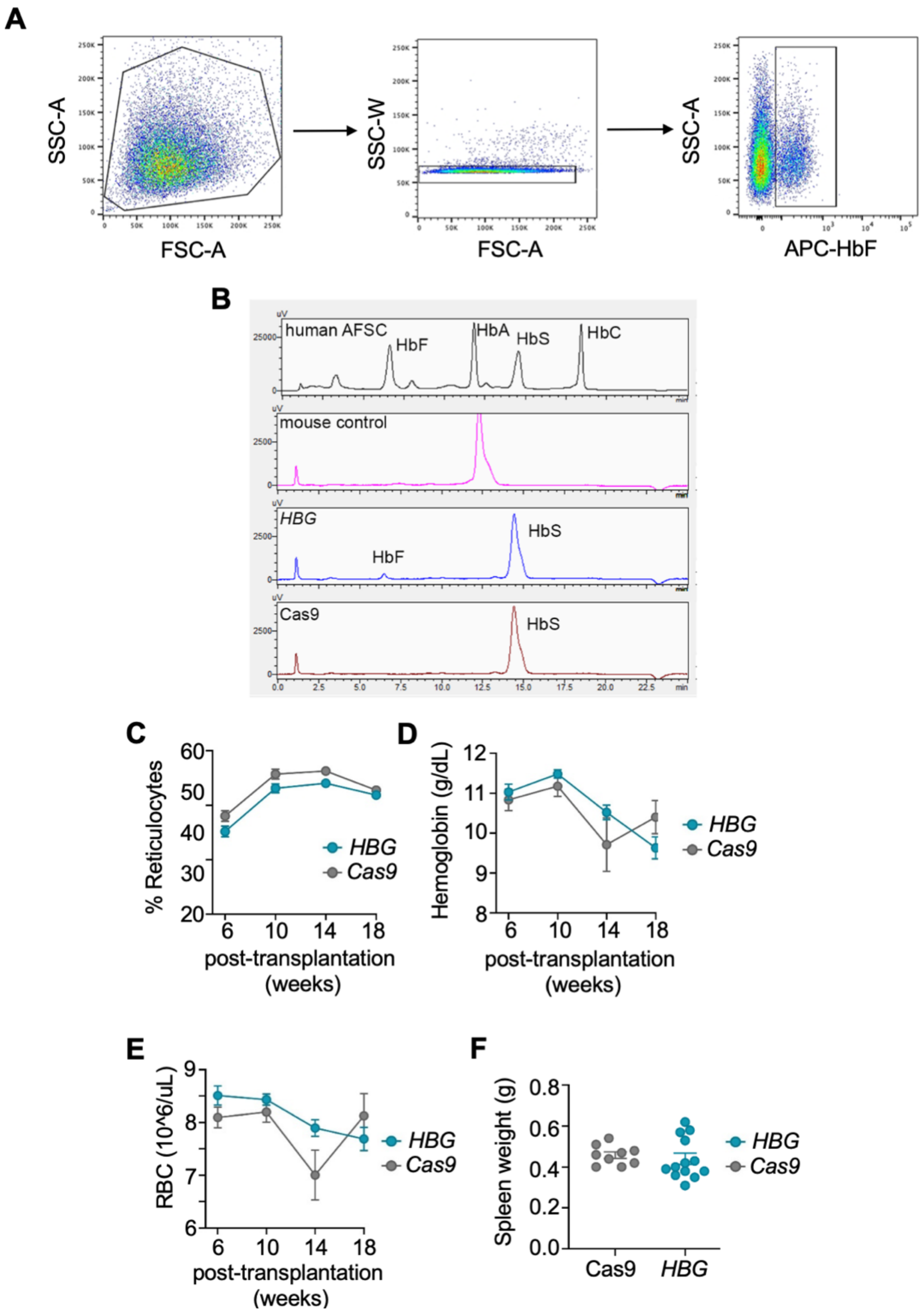

Disease Models & Mechanisms • Supplementary information

Fig. S7 (related to Figure 6). Cas9-mediated disruption of the γ-globin promoter BCL11A binding site in Townes mouse HSCs induces sub-physiological levels of HbF and fails to correct SCD phenotypes. Lin^- bone marrow HSPCs from Townes SCD mice were electroporated with RNP targeting the human *HBG1* promoter or Cas9 alone, transferred into lethally irradiated C57BL/6 hosts, and analyzed serially, beginning at six weeks post-HSCT. **(A)** Gating strategy for flow cytometry determination of fetal hemoglobin (HbF)-immunostaining RBCs ("F-cells). **(B)** Representative ion exchange high performance liquid chromatography profile from hemolysate at 14 weeks post-HSCT. **(C)** Percentage of reticulocytes versus time after HSCT. **(D)** Blood hemoglobin levels (g/dL) versus time after HSCT. **(E)** Peripheral blood RBC counts versus time after HSCT. **(F)** Spleen weights at necropsy performed at 18 weeks. Graphs in panels C-F show the mean ± SD of 2 biological replicate experiments consisting of 13 RNP-edited mice and 9 Cas9 mice total. No significant differences were observed between the two groups analyzed in panels C-F (t-test).

Disease Models & Mechanisms • Supplementary information

Table S1. PCR primer pairs used in this study

Gene or Locus	Species	Model	Application	Primer Seq 5’ to 3’	Size (bp)
Hbb-b1.F	Mouse	Berkeley	Genotyping	AAAGGTGAACTCCGATGAAGTTGG	442
Hbb-b1.R				ATAGGAAGGTTGAGCAGAATAGCC	
HBB.F	Human	Berkeley	Genotyping	ACATTTGCTTCTGACACAACTGTG	284
HBB.R				ACCACCAGCAGCCTAAGG	
Common	Mouse	Townes	Genotyping	TTGAGCAATGTGGACAGAGAAGG	--
Human WT	Human	Townes	Genotyping	GTTTAGCCAGGGACCGTTTCAG	320
Human SCD	Human	Townes	Genotyping	AATTCTGGCTTATCGGAGGCAAG	250
Mouse WT	Mouse	Townes	Genotyping	ATGTCAGAAGCAAATGTGAGGAGCA	432
MinION.F	Human	Townes	MinION seq	TTTGGCTCCAGGCACATTCCCACATCAAGATAAAGAGAAAC	~18.5kb mWT
MinION.R				AGTGGGTTGCATGGGTTCCTTACACACTTTGCA	
HBA1.1.F	Human	Berkeley	TLA seq	GCTCTGCCCAGGTTAAGG	242
HBA1.1.R				GTTGGTCTTGTCGGCAGG	
HBA1.2.F	Human	Berkeley	TLA seq	CCAAATACCGTTAAGCTGGA	265
HBA1.2.R				CTTGAAGTTGACCGGGTC	
HBBandHBG.1.F	Human	Berkeley	TLA seq	TATCAGAATGGCCCTAGTCT	751
HBBandHBG.1.R				ACTTCCGCAGAACACTTTAT	
HBBandHBG.2.F	Human	Berkeley	TLA seq	TTGTCTAAGTTGCCTCGAGA	575
HBBandHBG.2.R				CGTCTTCAGCCTACAACATAC	
MiniLCR.1.F	Human	Berkeley	TLA seq	CTCAGCCACCCTAATAGC	75
MiniLCR.1.R				GGACAGAGCACATTATAATTAAC	
MiniLCR.2.F	Human	Berkeley	TLA seq	TGTCTATATGGGTCGTTGTG	130
MiniLCR.2.R				AAAGACAAGCACGTGGAC	
HBGprom.DS.F	Human	Both	NGS	ACTGAATCGGAACAAGGCAAAGGCT	268
HBGprom.DS.R				ACCCATGGCGTCTGGACTAGGAGCT	
mROSA26.DS.F	Mouse	Both	NGS	TTCTGGGAGTTCTCTGCTGC	344
mROSA26.DS.R				TGGAAAATACTCCGAGGCGG	
mCdkn1a.F	Mouse	Both	qPCR	AGCCTGACAGATTTCTATCACTCC	24
mCcdkn1a.R				TGGGCACTTCAGGGTTTTCT	
mGAPDH.F	Mouse	Both	qPCR	GTGTTCCTACCCCCAATGTG	110
mGAPDH.R				AGGAGACAACCTGGTCCTCA	

Disease Models & Mechanisms • Supplementary information

Table S2. Bacterial artificial chromosome (BAC) probes used for fluorescent in situ hybridization

BAC Probe	Species	Model	Target	Location	Coordinates
RP23-308J23	Mouse	Berkeley	TIS	Centromeric to 1A5	mchr1:26,106,021-26,275,300
RP24-347O20				Telomeric to 1A5	mchr1:26.305.166-26,440,332
RP23-25C20	Mouse	Townes	Knock-In	Centromeric to 7E5	mchr7:103,573,095-103,768,675
RP23-367M2				Telomeric to 7E5	mchr7:103,894,127-104,083,626
RP23-239C8	Mouse	Both	*Rosa26* locus	Centromeric to 6E3	mchr6:112,778,187-113,016,809
RP23-33G8				Telomeric to 6E3	mchr6:113,106,213-113,297,830
RP23-223M9	Mouse	Both	*MYBL1* locus	mchr1 control probe	mchr1: 9,641,212-9,819,361 (GRCm39)
RP11-622D14	Human	Both	*HBB* locus	Complete *HBB* locus	chr11:5,218,918-5,406,749 hg19

Table S3. **Transgene copy numbers determined by droplet digital PCR.** SD, standard deviation

Target	Mean Copy Number	SD	n
HBA1	9.262	0.268	6
HBB	21.685	1.477	6
HBG2	26.590	0.302	6
Mini-LCR	3.942	0.092	6

Disease Models & Mechanisms • Supplementary information
